# Faecal amino acids stability: investigating optimal sampling conditions for analysis

**DOI:** 10.1007/s11306-025-02279-3

**Published:** 2025-06-14

**Authors:** Roza C. M. Opperman, Eva Vermeer, Sofie Bosch, Tim G. J. de Meij, Nanne K. H. de Boer, Eduard A. Struys

**Affiliations:** 1 Department of Gastroenterology and Hepatology, Amsterdam University Medical Centre, Vrije Universiteit Amsterdam, Amsterdam, 1081 HV The Netherlands; 2 Amsterdam Gastroenterology Endocrinology Metabolism (AGEM) Research Institute, Amsterdam University Medical Centre, Amsterdam, The Netherlands; 3 Cancer Centre Amsterdam (CCA) Research Institute, Amsterdam University Medical Centre, Amsterdam, The Netherlands; 4 Department of Paediatric Gastroenterology, Emma Children’s Hospital, Amsterdam University Medical Centre, Amsterdam, The Netherlands; 5 Amsterdam Reproduction & Development (AR&D) Research Institute, Amsterdam University Medical Centre, Amsterdam, The Netherlands; 6 Department of Laboratory Medicine, Amsterdam University Medical Centre, Amsterdam, The Netherlands

**Keywords:** Faecal amino acids, Targeted metabolomics, LC-MS/MS, Pre-analytical conditions, Sample stability

## Abstract

**Introduction:**

Faecal amino acids are promising non-invasive diagnostic biomarkers, but stability remains unclear.

**Objectives:**

This study examined whether storage conditions, sampling site within the sample, freeze-thaw cycles, and the OMNImet^®^·GUT device affect faecal amino acid concentrations.

**Methods:**

Faecal samples from three donors underwent various pre-analytical conditions. Amino acids were analysed through targeted liquid chromatography-tandem mass spectrometry.

**Results:**

Most amino acids remained stable across sampling sites and freeze-thaw cycles. Storage at -20 °C preserved integrity, whereas 4 °C and 20 °C storage led to variations. The OMNImet^®^·GUT device stabilised some amino acids but showed inconsistencies.

**Conclusion:**

Pre-analytical conditions influence faecal amino acid concentrations. Standardisation is essential for biomarker reliability.

**Supplementary Information:**

The online version contains supplementary material available at 10.1007/s11306-025-02279-3.

## Introduction

In recent years, the field of metabolomics has gained increasing scientific interest. In metabolomic analysis, small molecules can be analysed in several matrices, including plasma, urine, and faeces (Nicholson, & Lindon, 2008). Multiple studies have demonstrated the potential of metabolomics as non-invasive biomarkers for the detection and monitoring of a wide range of diseases, such as diabetes, cardiovascular and liver diseases, and various malignancies (Gowda et al., 2008). In gastroenterology, faecal metabolomics is a rapidly evolving field, showing promise in several gastrointestinal disorders such as colorectal cancer (CRC), inflammatory bowel disease (IBD), and disorders linked to a disturbed gut-brain interaction (Jagt et al., 2022b). Faecal metabolomics offers a unique advantage over direct microbiome profiling, as it more closely relates to gut microbiome activity and function. This makes metabolomics a complementary and potentially more reliable tool to understand gut physiology and pathophysiology in the context of disease. An altered gut microbiome composition is associated with these gastrointestinal diseases and may influence metabolic processes of the host, leading to changes in faecal metabolites, including amino acids (Bosch et al., 2020; Zierer et al., 2018). Whether microbiome changes drive metabolism in these disorders or vice versa remains unclear. Amino acids are involved in inflammation, immune regulation, microbial interactions, and metabolic pathways (Jagt et al., 2022a). Changes in amino acid concentrations may reflect gut microbiome activity and function. Bosch et al. presented a specific faecal amino acid panel that could detect CRC and adenomas with an accuracy of 79–98% (Bosch et al., 2022a, b). Moreover, faecal amino acid profiling holds promise for detecting IBD in children with an accuracy of 82% (Bosch et al., 2018; Jagt et al., 2022a). Other faecal metabolites that have shown potential as diagnostic or monitoring tools in gastrointestinal disease include short-chain fatty acids, bile acids, and volatile organic compounds (Jagt et al., 2022b). However, many of these are more volatile or have shown less consistent results across studies. Amino acids offer a relatively robust profile and are biochemically well-characterised, allowing for targeted and reproducible analysis. Their involvement in core physiological and immunological pathways further supports their relevance as biomarkers in gastrointestinal disease.

Faeces is a readily accessible matrix for studying gastrointestinal conditions as it provides an accurate reflection of intestinal health and disease. However, it is also highly complex, as its dry weight is composed of approximately 60% bacteria, with even a single gram containing roughly one billion bacteria (Sender et al., 2016; Stephen & Cummings, 1980). These bacteria are involved in various metabolic processes, but their composition and functionality can change once outside the body. Some may not survive, while others may adapt to different environmental circumstances (Ben-Amor et al., 2005). Notably, the faecal microbiome composition shifts after excretion due to exposure to oxygen, which particularly affects anaerobic species that thrive in the oxygen-deprived environment of the gut (Gorzelak et al., 2015). This can lead to the selective loss of certain bacteria and alterations in microbial interactions, potentially impacting downstream microbiome and metabolomic analyses. Therefore, ensuring that the measured analytes in faeces accurately represent the conditions at the moment of sampling is crucial.

Knowledge regarding the stability of faecal amino acids, a promising non-invasive biomarker, remains limited. Investigating the stability and optimal sampling methods of faecal amino acids is essential for establishing a standardised protocol for their analysis. Standardisation will enhance the consistency and comparability of research findings, and facilitate the broader application of faecal amino acid analysis as a diagnostic tool in clinical practice.

The aim of this study was to investigate the effect of various pre-analytical conditions on the composition of amino acids in faecal samples of healthy donors. The assessed conditions included storage duration and temperature, number of freeze-thaw cycles, the use of the OMNImet^®^·GUT sampling device, and variability across different locations within the faecal sample.

## Methods

### Sample collection

**Fig. 1:**
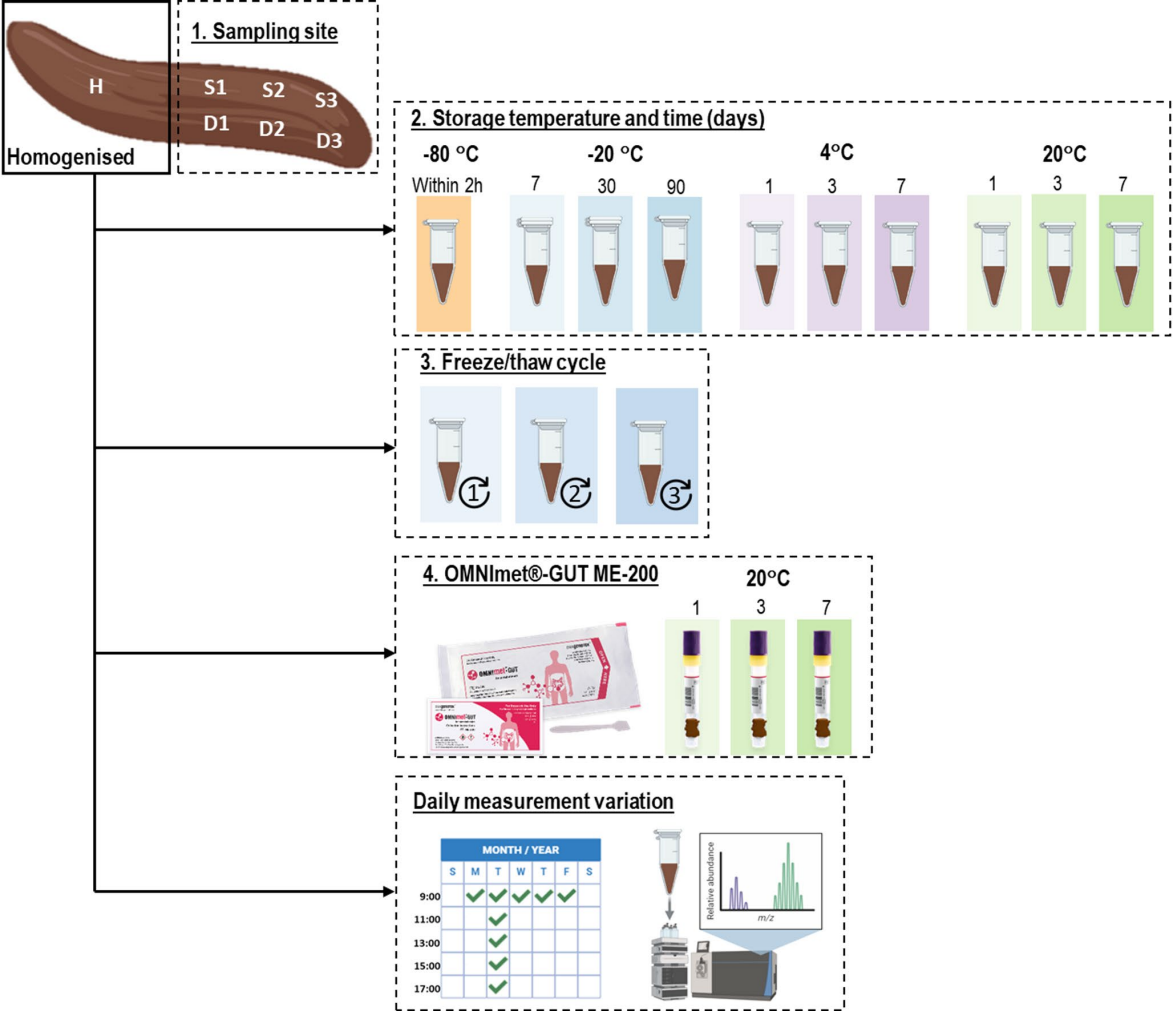
Schematic overview of pre-analytical conditions tested for all three healthy donors.* D1* deep location 1,* D2* deep location 2,* D3* deep location 3,* H* homogenised,* S1* superficial location 1,* S2* superficial location 2,* S3* superficial location

Faecal samples were collected from three healthy donors, each providing samples in seven separate containers (Faeces tube 76 × 20 mm, Sarstedt, Nümbrecht, Germany), according to the predefined sampling sites within the faecal sample. One sample was homogenised in 2 mL of distilled water, and vortexed for 5 min, serving as the reference. The remaining six samples were left unhomogenised to represent different sampling sites within the sample. All samples were divided into subsamples of 300 mg within two hours after sampling, before being subjected to the pre-analytical conditions. For each condition, experiments were conducted in duplicate per subject, resulting in six samples per condition. Following exposure to the pre-analytical conditions, samples were directly stored at -80 °C until further preparation for targeted amino acid analysis. It is assumed that in fresh-frozen faeces stored at -80 °C, no further metabolic processes occur that could alter the metabolite composition (Zouiouich et al., 2023). Freshly frozen homogenised faeces served as reference samples for comparison to each pre-analytical condition. Figure 1 depicts the various conditions assessed.

#### Sampling site

To assess the variability across different locations within the faecal sample, samples were collected from three different sites (both superficial and deep) in each stool sample, resulting in six distinct sampling sites.

#### Daily measurement variation

The precision of the analytical method was evaluated by calculating the coefficient of variation (CV%) for both intra- and inter-day variation. Intra-day variation was assessed by performing 5 repeated targeted measurements within a single analytical run. Inter-day variation was evaluated across 4 independent runs performed on different days.

#### Storage time and temperature

The impact of various storage temperatures and durations was evaluated. Separate samples were stored at home freezer temperature (-20 °C) for 7, 30, and 90 days, at refrigerator temperature (4 °C) for 1, 3, and 7 days, and at room temperature (20 °C) for 1, 3, and 7 days.

#### Freeze-thaw cycles

Samples were subjected to one, two, or three additional freeze-thaw cycles to evaluate the effect on amino acid concentrations. A single freeze-thaw cycle involved thawing at room temperature for 10 min, followed by re-freezing at -80 °C for 10 min.

#### OMNImet^®^·GUT sampling devices

OMNImet^®^·GUT sampling devices (ME-200, DNA Genotek, Inc.) were additionally assessed. Six devices were available for this evaluation. The devices were used according to the manufacturer’s instructions with homogenised stool samples from subjects 2 and 3. To evaluate the effects of varying storage durations at room temperature, one kit was stored immediately at -80 °C, while the other kits were kept at room temperature for three and seven days, respectively.

### Sample preparation

The sample preparation procedure involved homogenising, and, to minimise the effect of differences in faecal water content, freeze-drying. This comprised the addition of 1000 µL of distilled water to 300 mg (+-10%) of faeces, which was then homogenised by using a vortex for 1 min. Samples were subsequently transferred to a 100 mL round-bottom glass flask (Lenz, Wertheim, Germany). Using dry ice and isopropanol, a layer of frozen sample was formed inside the flask, followed by freeze-drying at -80 °C for 6 h (Christ Alpha 2–4). The remaining residue was collected and stored at -80 °C until further processing. After freeze-drying all samples, 5–6 mg of dry residual matter was mixed with distilled water to achieve a faeces-to-water ratio of 100 mg: 5 mL, and subsequently filtered to remove solid particles and fibres.

### Targeted amino acid analysis

Amino acid analysis was conducted using stable-isotope dilution liquid chromatography-tandem mass spectrometry (LC-MS/MS). In total, 10 µL of sample was mixed with 10 µL of internal standard mixture (ChromSystems), consisting of stable isotope-labelled internal standard, for all the individual amino acids measured. To derivatise the amino acids, 100 µL of borate buffer (65 mM, pH 11) and 100 µL of FMOC-Cl in acetone (15 mg / 10 ml) were added and mixed. Separation was achieved using a Waters BEH C18 UPLC column (1.7 μm particle size) on a Vanquish UPLC system (Thermo Scientific), coupled to a TSQ Quantiva tandem mass spectrometer (Thermo Scientific) with an electrospray ionisation source. A 10 µL amino acid panel calibrator (Chromsystems), taken into preparation as described for the processed faecal extract, served as quantitative reference. The amino acids mixture consisted of twenty unique amino acids previously identified as relevant for detecting gastrointestinal diseases, including alanine, α-amino adipic acid, citrulline, glutamate, glutamine, glycine, histidine, isoleucine, leucine, lysine, methionine, ornithine, phenylalanine, proline, serine, taurine, threonine, tryptophan, tyrosine, and valine (Bosch et al., 2018).

### Statistical analysis

Statistical analyses were performed in R Studio (version 4.2.1.). For all variables, the percentage concentration differences relative to the reference were calculated, except for the OMNImet^®^·GUT sampling devices. As the precise chemical composition of the OMNImet^®^·GUT medium was not disclosed, we chose to normalise the amino acid data by expressing each amino acid as a percentage of the total amino acid signal within the sample. We defined a ± 30% deviation from the reference as an indicative threshold to distinguish relevant changes from normal technical variability. This cut-off was used as a visual and interpretative aid, rather than a strict statistical criterion. While no universal threshold exists, a ± 30% deviation from reference is frequently an accepted range for variation in metabolomics studies involving complex biological matrices (Neuberger-Castillo et al., 2021). The coefficient of variation (CV) was calculated by dividing the standard deviation (SD) by the mean and multiplying the result by 100%.

## Results and discussion

First, the analytical precision of the LC-MS/MS method was evaluated. Inter-day CVs for the quantified metabolites were < 15% for 16 out of 20 amino acids. The amino acids valine (CV 18%), α-aminoadipic acid (CV 20%), tyrosine (CV 35%) and taurine (CV 69%) showed higher variability. Intra-day CVs ranged from 2 to 137%, with 18 out of 20 amino acids having a CV < 15%. The amino acids tyrosine (CV 19%) and taurine (CV 137%) showed higher variability. The high variability observed for taurine is partly due to the fact that its concentration was zero in 3 out of 8 measurements, which affects the reliability of the CV calculation. Consequently, taurine was excluded from all further analyses due to inconsistent results. The 35% inter-day CV of tyrosine may be explained by low signal intensities in a subset of samples, which can lead to increased variability in peak integration during quantification. Accordingly, some degree of analytical variability in tyrosine measurements should be considered when interpreting the results.

To evaluate the impact of pre-analytical conditions on amino acid concentrations, amino acids were analysed in stool samples from three healthy donors exposed to various conditions. First, the variability across different locations within the faecal sample was examined. In general, concentrations across different sampling sites remained within ± 30% of the reference value, based on the homogenised faecal sample (**Figure ****S1**). However, several outliers were observed. These outliers exhibited a consistent pattern across multiple measured amino acids, suggesting that the variability may be related to a common factor within the faecal matrix at the specific sampling site. Despite the complex and highly heterogeneous composition of faeces, the findings indicate that amino acid concentrations are generally stable across different sampling sites. In contrast, Gratton et al. evaluated faecal homogeneity and observed statistically significant differences in metabolome profiles across sampling sites (Gratton et al., 2016) However, their analysis included 506 metabolites, and the specific contribution of amino acids within this broader metabolite pool was not clearly defined. The current study focuses specifically on amino acids, providing a more targeted evaluation of their stability across sampling sites. In line with the findings of Santiago et al. on the microbiome, which demonstrated insignificant differences in species composition across different stool regions, this study shows that amino acid concentrations exhibit variability within acceptable limits for the majority of amino acids (Santiago et al., 2014).

**Fig. 2:**
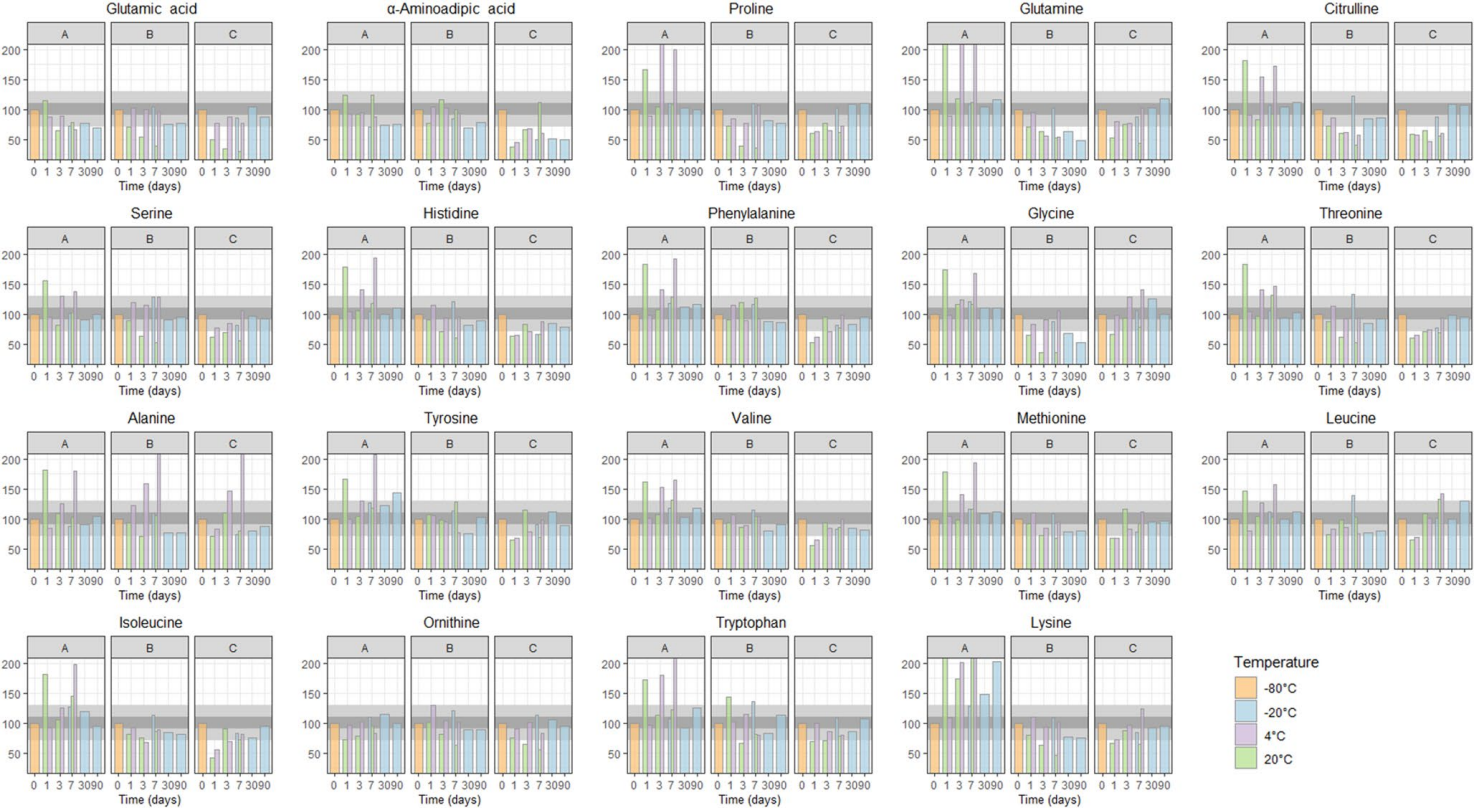
Relative faecal amino acid concentrations across the different storage durations and temperatures. Bar plots showing the relative concentrations of faecal amino acids across different storage durations and temperatures. Each plot represents a specific amino acid. Storage times in days are depicted along the x-axis, and the relative amino acid concentrations are shown on the y-axis. Colors correspond to the different storage temperatures as detailed in the legend. The dark gray shaded area represents a deviation of approximately ± 10% from the reference sample, while the lighter gray area indicates a deviation of around ± 30%

Second, the influence of storage temperature and duration were assessed and compared to the homogenised sample stored at -80 °C within 2 h of collection (Fig. 2). At room temperature (20 °C) and refrigerator temperature (4 °C), variability was observed across storage durations (1, 3, and 7 days), as well as between individual amino acids and donors. These results suggest that potential metabolic processes persisting at these temperatures are both individual- and amino acid-dependent. Gratton and colleagues observed that freezing samples at -20 °C immediately altered the metabolic composition of crude faecal samples, resulting in elevated amino acid concentration (Gratton et al., 2016). However, in the current study, samples stored at -20 °C for 7 days, 1 month, and 3 months showed concentrations predominantly within 10% deviation of the reference values, with deviations generally remaining within 30%. This suggests that, in addition to storage at -80 °C, storage at -20 °C is also effective in preserving amino acid concentrations for up to 3 months.

Regarding the effect of up to three freeze-thaw cycles, amino acid concentrations generally deviated within ± 30% from the reference sample, without showing a consistent trend (**Figure ****S2**). Only in donor 2 (green), a clear decrease in amino acid concentrations was observed for all amino acids after the second freeze-thaw cycle, which may be more related to the sample processing rather than the freeze-thaw effect itself. This is further supported by the observation that concentrations increased again after the third cycle. A previous study reported an increase in valine, glycine, alanine, phenylalanine, and tyrosine after freeze-thawing, though the corresponding CV values were not specified (Gratton et al., 2016). In the current study, samples were exposed to room temperature for a maximum of 5 min, until the sample thawed, before being refrozen. In contrast, the referenced study subjected samples to 4 °C storage for 24 h, followed by 5 h at room temperature, resulting in longer exposure to higher temperatures and increased susceptibility to metabolic processes (Gratton et al., 2016). Overall, no clear decreasing or increasing trends were observed across the number of cycles in the current study, which makes it more likely that the observed variability is attributable to inter- and intra-day variation.

Finally, the stability of amino acids in faecal samples collected using the OMNImet^®^·GUT sampling device was assessed. The OMNImet^®^·GUT device facilitates homogenisation and metabolite stabilisation at room temperature up to 7 days (**Figure S3**). This was tested in two out of three donors due to insufficient sample from the third. Previous studies by the manufacturer in collaboration with Metabolon demonstrated strong correlations between fresh-frozen faeces and samples stored in the OMNImet^®^·GUT device at room temperature for 0, 1, 4, and 7 days, as measured by ultra-high performance LC-MS/MS (UHPLC-MS/MS) (De Bruin et al., 2020). In the current study, while isoleucine showed significant variability, lysine, methionine, alanine, serine, and phenylalanine remained stable up to day 7. These findings support the potential of the OMNImet^®^·GUT device for stabilising faecal amino acids, making it a promising tool for both research and potentially clinical applications involving validated faecal amino acid biomarkers.

We acknowledge the limitation of our study’s small sample size. This number was chosen to maintain feasibility, given the range of pre-analytical conditions tested per donor in duplo. Our analyses primarily focused on within-individual comparisons, allowing us to assess the relative effects of each condition. While this design enabled a detailed evaluation of condition-related effects, the limited number of donors means that generalisability to the wider population remains to be confirmed.

## Conclusion

In conclusion, the impact of pre-analytical conditions on faecal amino acid concentrations is both amino acid-specific and subject-dependent. Taurine exhibited considerable variability and was often undetectable, limiting its potential as a biomarker. Strict standardisation would be necessary for any potential diagnostic application. Although faecal amino acid concentrations between different sampling sites within the faecal sample generally remained within ± 30% deviation of the reference, outliers could influence results. Therefore, we recommend homogenisation of faecal samples prior to amino acid analysis. Regarding storage conditions, faecal amino acid concentrations remained stable at -20 °C, whereas storage at room temperature or refrigeration resulted in considerable variability. The number of freeze-thaw cycles had minimal impact on most amino acids. Finally, this study indicates that sampling devices such as the OMNImet^®^·GUT may have the potential to stabilise amino acids at room temperature, facilitating home sampling. However, further optimisation is required for specific amino acids. This study provides a foundation for establishing optimal pre-analytical conditions for faecal amino acids that may serve as potential non-invasive biomarkers in clinical practice.

## Electronic supplementary material

Below is the link to the electronic supplementary material.

Supplementary file1 (DOCX 426 KB)

## Data Availability

After completion and publication of the trial coded data will be available from the corresponding author on reasonable request.
