# Supplementary material for: Faecal amino acids stability: investigating optimal sampling conditions for analysis
Source: Metabolomics. 2025 Jun 14;21(4):81. doi: 10.1007/s11306-025-02279-3 (PMC12167247; doi:10.1007/s11306-025-02279-3)
Supplement: Supplementary file 1 — Supplementary file1 (DOCX 426 KB) [file 11306_2025_2279_MOESM1_ESM.docx]

Supplementary material

Faecal Amino Acids Stability: Investigating Optimal Sampling Conditions for Analysis

Roza C.M. Opperman^1,2,3*^, Eva Vermeer^2,4,5*^, Sofie Bosch^1,2^, Tim G.J. de Meij^2,4^, Nanne K.H. de Boer^1,2^, Eduard A. Struys^6^

* Contributed equally (shared first authorship)

^1^ Department of Gastroenterology and Hepatology, Amsterdam University Medical Centre, Vrije Universiteit Amsterdam, Amsterdam, The Netherlands.

^2^ Amsterdam Gastroenterology Endocrinology Metabolism (AGEM) Research Institute, Amsterdam, The Netherlands.

^3^ Cancer Centre Amsterdam, research program, Amsterdam, The Netherlands.

^4^ Department of Paediatric Gastroenterology, Emma Children’s Hospital, Amsterdam University Medical Centre, Amsterdam, The Netherlands

^5^ Amsterdam Reproduction & Development (AR&D) Research Institute, Amsterdam University Medical Centre, Amsterdam, The Netherlands

^6^ Department of Laboratory Medicine, Amsterdam University Medical Centre, Amsterdam, The Netherlands

**Keywords**: faecal amino acids, targeted metabolomics, LC-MS/MS, pre-analytical conditions, sample stability

**Corresponding authors**:

R.C.M. Opperman, MD

Department of Gastroenterology and Hepatology

Amsterdam University Medical Centre, Vrije Universiteit Amsterdam

1081 HV Amsterdam

The Netherlands

ORCID: 0000-0002-7279-0940

Email: [r.opperman@amsterdamumc.nl](mailto:r.opperman@amsterdamumc.nl)

E. Vermeer, MD

Department of Paediatric Gastroenterology

Emma Children’s Hospital, Amsterdam University Medical Centre

1105 AZ Amsterdam

The Netherlands

ORCID: 0009-0009-4335-0100

Email: [e.vermeer@amsterdamumc.nl](mailto:e.vermeer@amsterdamumc.nl)

**TABLE OF CONTENTS**

| **Topic** | **Page** |
| --- | --- |
| **Supplemental figure 1.** Relative faecal amino acid concentrations across the different sampling sites within the faecal sample. | 3 |
| **Supplemental figure 2.** Relative faecal amino acid concentrations across the different storage durations and temperatures. | 4 |
| **Supplemental figure 3.** Relative faecal amino acid concentrations across the different numbers of freeze-thaw cycles. | 5 |
| **Supplemental figure 4.** Relative faecal amino acids concentrations for the OMNImet®·GUT sampling device. | 6 |


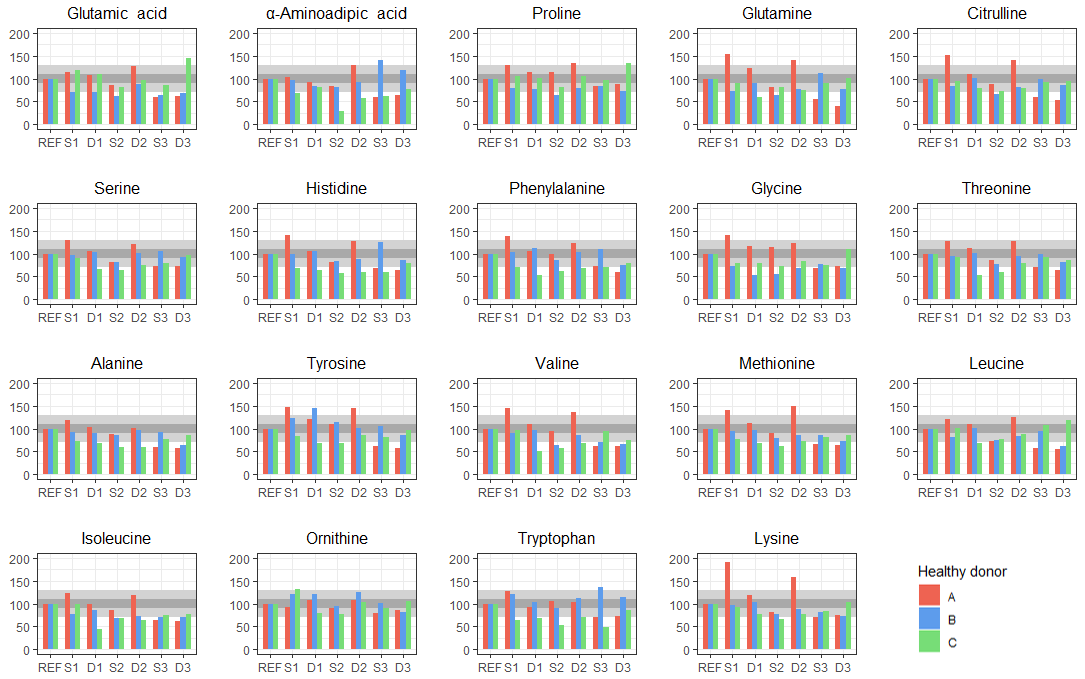


**Supplemental figure 1**. Relative faecal amino acid concentrations across the different sampling sites within the faecal sample.

Barplots displaying the relative concentrations of faecal amino acids across different sampling sites. Each plot represents a specific amino acid. The x-axis indicates the sampling site, while the y-axis shows the relative amino acid concentrations. Colors correspond to the different healthy donors as detailed in the legend. The dark gray shaded area represents a deviation of approximately ±10% from the reference sample, while the lighter gray area indicates a deviation of around ±30%. Abbreviations: D – deep location, S – superficial location.


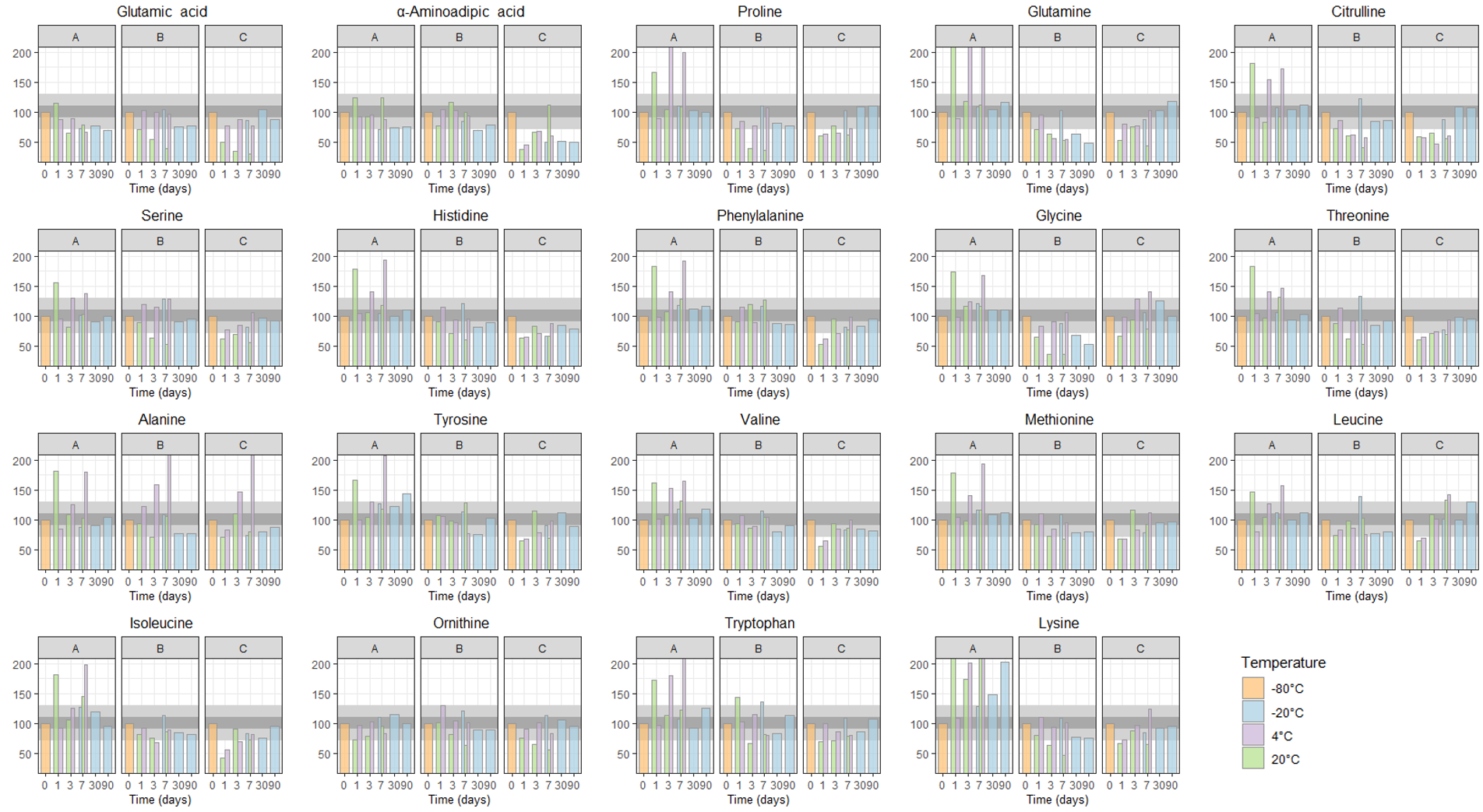


**Supplemental figure 2.** Relative faecal amino acid concentrations across the different storage durations and temperatures.

Barplots showing the relative concentrations of faecal amino acids across different storage durations and temperatures. Each plot represents a specific amino acid. Storage times in days are depicted along the x-axis, and the relative amino acid concentrations are shown on the y-axis. Colors correspond to the different storage temperatures as detailed in the legend. The dark gray shaded area represents a deviation of approximately ±10% from the reference sample, while the lighter gray area indicates a deviation of around ±30%.


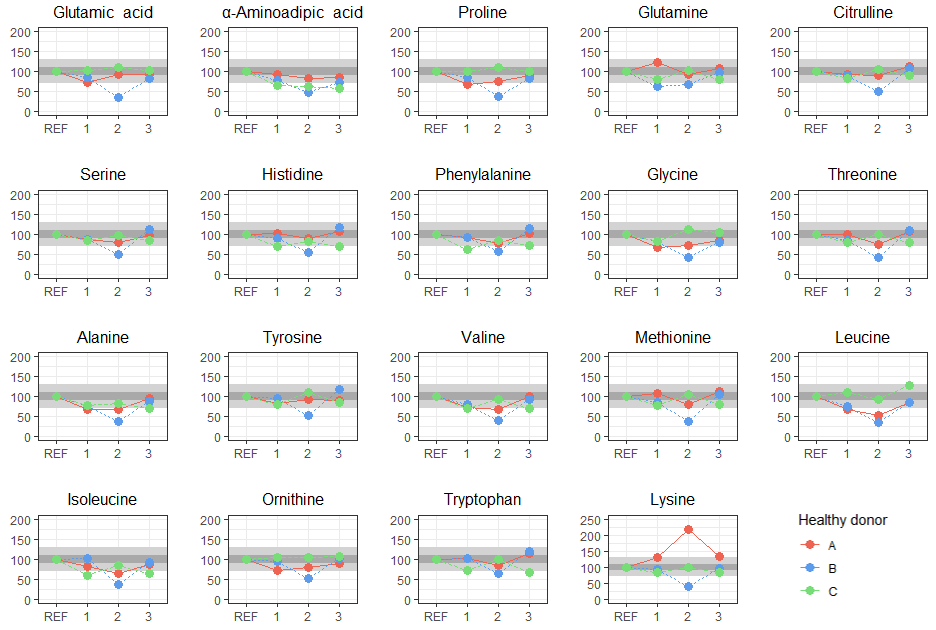


**Supplemental figure 3**. Relative faecal amino acid concentrations across the different numbers of freeze-thaw cycles.

Lineplots displaying the relative concentrations of faecal amino acids across the different numbers of freeze-thaw cycles. Each plot represents a specific amino acid. The x-axis indicates the freeze-thaw cycles, while the y-axis shows the relative amino acid concentrations. Colors correspond to the different healthy donors as detailed in the legend. The dark gray shaded area represents a deviation of approximately ±10% from the reference sample, while the lighter gray area indicates a deviation of around ±30%.


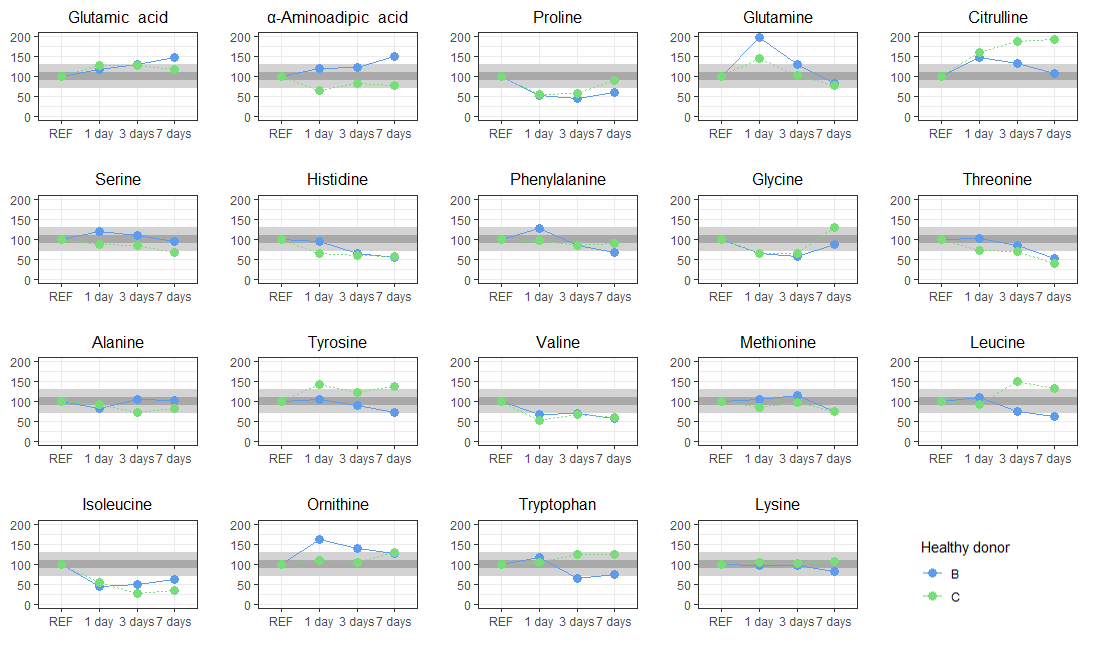


**Supplemental figure 4.** Relative faecal amino acid concentrations for the OMNImet®·GUT sampling device.

Lineplots displaying the relative concentrations of faecal amino acids across different storage times. Each plot represents a specific amino acid. The x-axis indicates the days of storage, while the y-axis shows the relative amino acid concentrations. Colors correspond to the different healthy donors as detailed in the legend. The dark gray shaded area represents a deviation of approximately ±10% from the reference sample, while the lighter gray area indicates a deviation of around ±30%.
